# Intelligent Error Correction of College English Spoken Grammar Based on the GA-MLP-NN Algorithm

**DOI:** 10.1155/2021/7371416

**Published:** 2021-12-22

**Authors:** Yining Du

**Affiliations:** Department of Foreign Languages, Xi'an University of Finance and Economics, Xi'an, Shaanxi 710010, China

## Abstract

With the development of neural networks in deep learning, artificial intelligence machine learning has become the main focus of researchers. In College English grammar detection, oral grammar is the most error rate content. So, this paper optimizes MLP based on GA in the deep learning neural network and then studies the intelligent image correction of College English spoken grammar. The main direction is to discuss and analyze GA-MLP-NN algorithm technology first and then predict the error correction model of spoken language grammar by combining the optimized algorithm. The results show that GA-MLP-NN provides excellent accuracy for the prediction of the whole syntax error correction model. Then, the paper studies the deep learning technology to build an intelligent image error correction model of College English spoken grammar. The results show that the effect of intelligent correction of spoken grammar is very fast and accurate.

## 1. Introduction

With deep learning, neural networks and other research methods gradually become the focus of the development trend of intelligent image society [1]. Scientists in various countries have made countless achievements in this algorithm technology. Deep learning is based on many levels of processing calculation data to establish the model and ultimately achieve the prediction and analysis of the actual results [2, 3]. When the back propagation technology in the neural network is combined with the development of the artificial neural network, the traditional mode of connecting data calculation and optimization algorithm began to change [4]. Subsequently, the deep learning algorithm is proposed, which gradually becomes the representative of intelligent technology. Due to the continuous transformation and updating of various network structures, neural unit structures, and hierarchical structures, many new neural networks begin to emerge [5, 6]. Deep learning is the internal law and representation level of learning sample data. The information obtained in the learning process is very helpful to the interpretation of data such as text, image, and sound. Its ultimate goal is to make the machine have the ability to analyze and learn like human beings and be able to recognize characters, images, sounds, and other data. Deep learning is a complex machine learning algorithm, which has achieved far more results in speech and image recognition than previous related technologies [7]. The development of the deep learning algorithm also stimulates progress in the field of artificial intelligence, and the classified learning forms the basic machine learning content [8].

In the nature of deep learning supervision, the construction parameters and construction model of the network are obtained by network structure training based on the marked learning and training data [9]. The establishment of the network structure parameter model in the unsupervised nature of deep learning does not require the participation of the marked data [10]. The concept of deep learning comes from the research of the artificial neural network. Multilayer perceptron with multiple hidden layers is a deep learning structure. These three kinds of deep neural networks are applied in different fields and technologies [11]. The combination of the genetic algorithm and multilayer perceptron neural network is a hybrid method of DL and the metaheuristic algorithm [12]. Multilayer perceptron neural network is the earliest basic research result in the algorithm, and it is also the algorithm basis for deep learning neural network structure performance analysis and comparison [13, 14]. Other neural networks include the convolution neural network and the cyclic neural network. It is usually used in the field of computer vision pattern recognition detection, segmentation, tracking, quantization, and so on [15]. They can make the network model have memory ability, which is more suitable for data processing in the process of learning and training.

With the further development and use of research results, the problems of the deep learning neural network are gradually emerging [16]. The main problems are as follows: in the neural network, the amount of calculation data of multiple units is very large, and there are many variables that need to be involved in the calculation. For the use and acquisition of parameters in the whole network operation, it is usually difficult to select [17]. This complex structure often affects the accuracy of the final output data of the whole network model. Therefore, we should combine a variety of neural networks as the basis of the construction algorithm of the system model [18].

The innovation of this paper is to optimize the MLP based on the Genetic Algorithm in the in-depth learning neural network and study the intelligent image correction of College English spoken grammar. The main direction is to first discuss and analyze the GA-MLP-NN algorithm technology and then predict the error correction model of oral grammar combined with the optimization algorithm. The results show that GA-MLP-NN provides good accuracy for the prediction of the whole syntax error correction model. Compared with the traditional multilayer perceptron prediction, the optimization algorithm significantly improves the operation efficiency of the model and shortens the prediction time.

This paper is divided into three parts. The first part is a brief introduction to the current development of deep learning and neural networks at home and abroad. The second part is based on GA-MLP-NN neural network technology research and its role in the oral grammar error correction model, as well as the deep learning algorithm in College English spoken grammar intelligent image error correction model construction technology research. The third part is the result analysis of GA-MLP-NN technology and the model result analysis of deep learning technology in oral grammar intelligent image error correction research.

## 2. The Related Works

Artificial neural network is an important part in the field of intelligence. In the technical requirements of developing machine learn Artificial Intelligence, the training is carried out according to the neural network [19]. The first neural network formed at the beginning of the period was an artificial mathematical model, and then, the back propagation (BP) algorithm was optimized to form a new training neural network. The problem of the deep learning neural network is that a large amount of learning and training data is needed as the basis for the construction of network structure [20]. Their requirements for accuracy and parameters are far from satisfying. In order to improve the defects of the deep learning single neural network, we need to use the genetic algorithm to optimize the structure of various neural networks [21].

After research, it is found that the genetic algorithm can not only find the weight of neural network connection but also be extended to find the whole neural network [22]. The researchers of the team of scientists have been working with excellent in-depth learning experts from all over the world. The intelligent image recognition technology based on the artificial neural network is studied. The development of this technology has completely changed the structural framework of traditional recognition technology [23].

Computer scientists conceived and studied the development of artificial intelligence [24]. Then, there appeared the principle of modern computer composition. Computer technology was used to compile and translate passwords, and it was not until the last century that computer technology was widely used to solve complex problems [25]. The researchers combine the genetic algorithm with the neural network to change the structure of neural units [26]. The neural network improves the performance of basic artificial intelligence.

The deep learning algorithm is also very advanced, and their AI institute can use supercomputers to perform the deep learning function. It can improve the learning and training speed of the convolutional neural network to 100 times. Then, according to the continuous in-depth research of neural networks, this technology is used in the automotive field, intelligent image recognition field, and so on [27].

In deep learning and neural networks, researchers have proposed a neural network structure based on genetic algorithm optimization [28]. In the existing technology, the MLP neural network structure of multilayer perceptron can be optimized according to the coding operation mode [29, 30]. The node quantity of the involute layer is represented by binary, and the weight gradient is calculated randomly to achieve the supervised operation of learning training [31]. The optimized neural network is widely used in various prediction models and simulation models.

Based on the analysis of the development of deep learning and neural network, this paper proposes a prediction model based on the GA-MLP-NN neural network. The final learning and training effect can improve the accuracy of the whole model.

## 3. Research on Intelligent Image Error Correction Technology of College English Oral Grammar Based on GA-MLP-NN Algorithm and Deep Learning

### 3.1. Research on Oral Grammar Error Correction Technology Based on GA-MLP-NN

**Figure 1:**
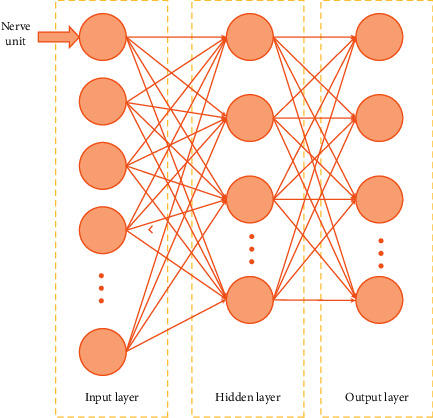
Framework of multilayer perceptron.

The artificial neural network system is a kind of network structure which can realize the output data of multiple nodes and solve practical problems. Both the human brain and the artificial neural network can process a lot of data and information. However, the specific operation mode of the neural network is different from that of human beings. We can apply a neural network to an intelligent image machine and use the linear regression method to make the computer learn the human computing process. There are many algorithms in the artificial neural network, and the MLP algorithm is one of them. Multilayer perceptron system is also called feedforward multilayer neural network structure. The whole frame is shown in Figure 1.

Multilayer perceptron neural network structure has excellent nonlinear operation ability. In the model learning training, the back propagation neural algorithm can improve the large error of the model in the input and output data. The mapping function of the whole MLP neural network can be reflected in the common processing of the whole data and achieve comprehensive optimization performance. But in the multilevel structure processing, the defects are also obvious. The most significant is that the computational efficiency is relatively low, and the number of nodes and the use of neural units in the gradually hidden layer exceed the overall variable parameters. This situation leads to slow processing in the process of learning and training, which requires high support computing ability. In the traditional multilayer perceptron model, the unit neural network can only use a single data for operation, and it cannot achieve the expected effect when a multilevel is carried out together.

**Figure 2:**
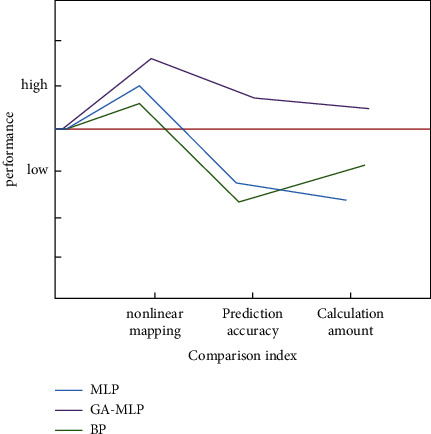
Performance comparison of MLP, GA-MLP, and BP algorithm.

In order to solve the problem of MLP, we propose a genetic neural network based on GA arithmetic. When different layers of the model work together, the improved neural network has a clear number of nodes. It can make the complex composition of the whole simulation system clear. According to the characteristics of the human brain, we add the glial neural network to the improved multilayer perceptron. In other words, the fading layer of the multilayer perceptron is connected with the glial network to form the model network after computer simulation and optimization. This method makes the MLP algorithm have better performance. The optimized GA-MLP-NN neural network can realize the point-to-point variable information data processing; that is, each data information is calculated as a separate real-time operation. It can realize a multidirectional calculation at the same time and improve the efficiency of the whole learning and training. The MLP algorithm of the single multilayer perceptron before and after optimization is compared with the optimized algorithm and the traditional BP algorithm. The comparison results are shown in Figure 2.

**Figure 3:**
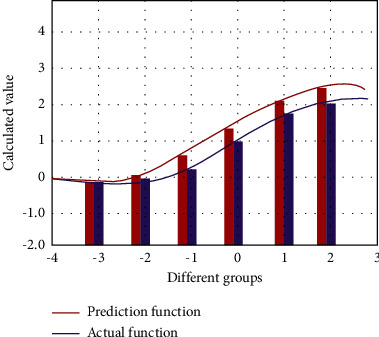
The actual and predicted change chart of *S*-type function.

In the process of correcting College spoken English grammar, it is necessary to test the performance of the whole model and the accuracy of the output data. In the face of more and more strict grammar rules, the detection content also needs to be more accurate. Therefore, we evaluate the performance of the whole model, and test and judge the accuracy. Firstly, the linear operation is carried out according to the linear regression algorithm to calculate the relationship between the number of grammar and the accuracy of error correction. According to the causality, it is transformed into a linear equation for calculation, and the final result is obtained by the least square method. According to the trend of the results, the error correction ability of the prediction model is detected. MLP neural network is a traditional front neural network, which can store and train data according to simulated brain activity. The optimized GA-MLP-NN algorithm can learn more complex structure processing methods. A progressive hidden layer unit neural network is used to carry out a multilevel accurate continuous operation with an *S*-type recursive function. It improves the problem that the traditional way of increasing the number of nodes and layers in the gradually hidden layer will lead to poor training results. According to the weight value matrix of each level, the *S*-type function is used, and the change of prediction function value before and after is shown in Figure 3.

The formula for calculating the weight of each level of nonlinear mapping is as follows:

(1) $$\begin{array}{c} A=f\left(W_{1}^{T}*X\right),\\ Y=f\left(W_{2}^{T}*A\right). \end{array}$$

The ultimate goal of training is to reduce the actual prediction error between input data and output data and gradually optimize the output data. In the process of learning and training, it is necessary to adjust the weight value as close to the function range as possible to achieve the purpose of optimal global solution. It is necessary to determine the output matrix of the gradually hidden layer:

(2) $$\begin{array}{c} {\left\|W_{2}^{T}*R-S\right\|}_{2}=\min. \end{array}$$

According to the above calculation, the minimum range solution of the gradually hidden layer can be obtained as follows:

(3) $$\begin{array}{c} {\left\|W_{2}^{T}*\Delta R-\left(S-W_{2}^{T}*A\right)\right\|}_{2}=\min. \end{array}$$

According to the transfer function, the parameter value of the matrix *R* is determined to be in a certain range. If it is beyond the expected range, the weight matrix needs to be changed, and the expected output value of the gradually hidden layer needs to be increased.

(4) $$\begin{array}{c} W_{T}=\left[W_{1}W\right],\\ X_{r}=\left[XR\right]. \end{array}$$

The model used in linear regression analysis is as follows:

(5) $$\begin{array}{c} Y=a+bX+\varepsilon . \end{array}$$

The formula includes the constant term, regression coefficient, and error number, that is, the influence of variables on the whole prediction process. The accuracy and variable parameter estimation are verified according to the statistical check, and the fitting degree and significance performance are evaluated according to the unified detection method. The fitting degree is the calculated value after the prediction of the sample data. The calculation formula is as follows:

(6) $$\begin{array}{c} \text{ESS}=\sum {\left({\overset{\wedge }{Y}}_{i}-\overset{-}{Y}\right)}^{2},\\ \text{RSS}=\sum {\left(Y_{i}-{\overset{\wedge }{Y}}_{i}\right)}^{2}. \end{array}$$

For the overall linear significant detection of variable parameters, the statistical calculation is required, and the calculation formula is as follows:

(7) $$\begin{array}{c} t-\frac{R}{\sqrt{\left(1-R^{2}\right)\left(n-2\right)}}. \end{array}$$

The GA-MLP-NN neural network model needs to process the test data in the College English oral error correction function. The whole multilayer perceptron first needs to solve the value of the input data and then brings the obtained parameter variables into the activation function. The activation function formula is as follows:

(8) $$\begin{array}{c} \varphi \left(v\right)=\tanh\left(v\right). \end{array}$$

The output value can be represented by the mathematical formula

(9) $$\begin{array}{c} \overset{\wedge }{y}=\tanh\left(d\sum_{d=1,n=1}^{n}w_{d}x_{n}\right). \end{array}$$

**Figure 4:**
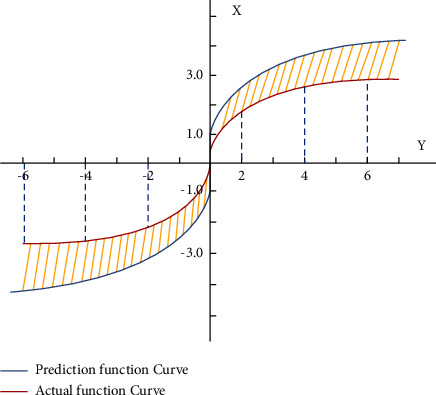
Function curve.

The comparison between the actual function curve and the predicted function curve is shown in Figure 4.

GA-MLP-NN is trained by adjusting the weight value and threshold parameter range, and the ultimate goal is to achieve the consistency or approximation between the actual output and the training sample data:

(10) $$\begin{array}{c} w_{j}^{k+1}=w_{j}^{k}+\beta \left(y_{i}-y_{i}^{k}\right)x_{ij}. \end{array}$$

**Figure 5:**
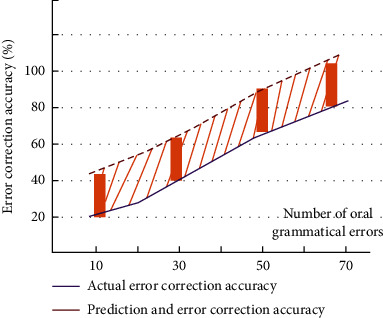
Comparison chart of GA-MLP-NN model to variables.

If the result is closer to the exact value than expected, you can continue to use the current weights and thresholds. On the contrary, if the prediction estimation is not accurate, the weight value and threshold need to be updated to adjust the accuracy. We choose two variables in the model of College English oral grammar error correction, including the number of grammar and the accuracy of error correction. The established comparison data are shown in Figure 5.

### 3.2. Research on Intelligent Error Correction Model of College English Oral Grammar Based on Deep Learning

**Figure 6:**
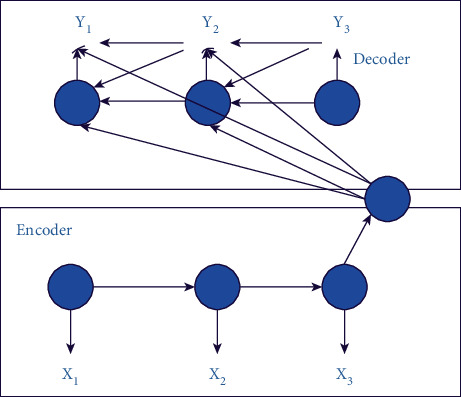
Content-based framework of seq2seq.

As an international language, English is widely used in daily life, work, and study. The use of English grammar should be standardized and accurate. Grammar, as an important part of actual semantics, is used in more and more ways due to the increasing number of learners. In daily communication, we usually use spoken English to express our ideas. How to use spoken English grammar intelligently to realize the function of error correction is very important. At present, with the development of machine learning language, speech recognition technology is also combined with an intelligent image model. However, none of the above situations can realize the intelligent image error correction of oral English grammar. So we study the use of deep learning and neural networks for machine learning training as an important way of machine training. In the nonlinear structural level, the deep learning algorithm can be more accurate by fast and effective accuracy learning for data analysis. We use deep learning combined with the algorithm structure of the neural network model to realize the research on the intelligent image error correction ability of oral English grammar. seq2seq is a generative model. At the beginning of its birth, it is mainly to solve the problem that RNN cannot handle indefinite length pairing. The structure of seq2seq can better deal with the output problems in some scenarios. The typical is the multistep prediction of time series prediction. There is a strong sequence correlation between tags. The structure of seq2seq can better deal with the output problems in some scenarios. Deep learning can record used data information and provide effective help in image processing, translation, and other functions. In deep learning, we mainly study the seq2seq model, whose coding and decoding functions can sort the neural network sequences. The content-based seq2seq framework is shown in Figure 6.

In the framework structure, according to the input queue to transform semantic rules and generate corresponding variables, it is necessary to determine the upper and lower time states of the fading state:

(11) $$\begin{array}{c} h_{t}=f\left(h_{t-1},x_{t}\right). \end{array}$$

The translation model variables of the fading situation in time are defined, in which the information and feature point data in the whole input data queue are replied:

(12) $$\begin{array}{c} C=q\left(h_{1},h_{2},h_{3},\ldots ,h_{t}\right). \end{array}$$

According to the decoding function of the model, the obtained variable statements are decoded, and the generated variables and output queues are used to predict the next error correction.

(13) $$\begin{array}{c} y_{t}=\arg\max P\left(y_{t}\right),\\ y_{t}=\prod_{t=1}^{T}p\left(y_{t}\;|\;\left\{y_{1},y_{2},\ldots ,y_{t-1}\right\},C\right). \end{array}$$

In the decoding process, we need to use the hidden calculation formula as follows:

(14) $$\begin{array}{c} h_{t}^{\prime }=f\left(h_{t-1}^{\prime },y_{t-1},C\right). \end{array}$$

In the whole model, the encoder queues the input data into a fixed range length. In the process of calculating variables, a lot of detailed information about data will be lost. The longer the grammatical rules in the whole English sentence, the more frames the information data lose. So we need to introduce an attention mechanism to solve this problem when we plan a deep learning model. In the whole framework of the seq2seq model, the information synchronization probability should be combined.

(15) $$\begin{array}{c} p\left(y_{i}|y_{1},\ldots ,y_{i-1},x\right)=g\left(y_{i-1},s_{i},c_{i}\right),\\ e_{tj}=a\left(s_{i-1},h_{j}\right),\\ a_{ij}=\frac{\text{exe}\left(e_{ij}\right)}{\sum_{K-1}^{T}\exp\left(e_{ik}\right)},\\ c_{i}=\sum_{j=1}^{T}a_{ij}h_{j}. \end{array}$$

**Figure 7:**
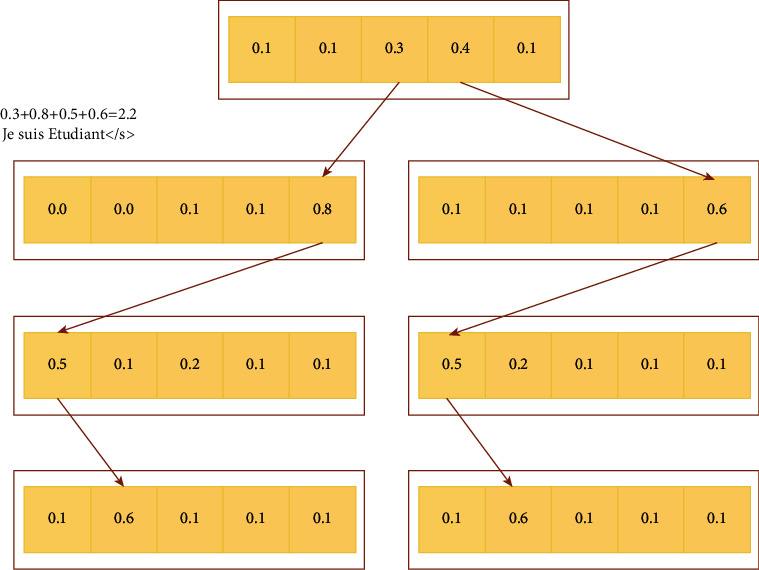
Breadth search output process.

The formula includes the output state variables of the hidden end in different time periods, as well as the error correction probability and statistics of different time nodes. In order to prevent the text data of the whole statement from being saved effectively, we will parse the improved framework. It is necessary to establish a prediction model for each time node for position prediction. In the face of a large number of spoken words in a large number of data sentences, it is easy to lead to improper storage and leakage. According to this situation, we use the search function implantation, and the output process of breadth search is shown in Figure 7.

According to the input data and semantic variables, the distribution of spoken words is analyzed, and then, the output state determination option is selected as the starting node of the next prediction. This kind of breadth output search method can solve the problem of space storage occupying the whole machine memory, but there are some rare words that cannot be effectively identified for the input high-level words. In the future, we will add a replica mechanism to the research model to supplement the database by copying high-end words and rare words, and finally solve the problem of nonrecognization. After improving the database support, we need to go through the following stages to analyze a whole spoken sentence.

Firstly, the whole sentence is truncated to segment the whole English sentence text. the original sentence is cut into separate sentences, such as punctuation. But this truncation method is easy to cause semantic errors. Then, the words in the grammar are split, and the segmentation needs to be trained according to the machine language learning function. Finally, the whole sentence is analyzed using semantic analysis, because the use of the English environment is more complex, so most of the translation will form a variety of word meanings. Therefore, whether the analysis of the whole word meaning is standardized and reasonable is an important factor for the effect of error correction.

## 4. Analysis of the Research Results of College English Oral Grammar Intelligent Image Error Correction Based on GA-MLP-NN Algorithm and Deep Learning

**Figure 8:**
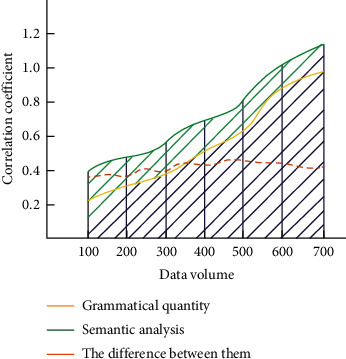
Correlation coefficient comparison.

Through the above research, we know that the accuracy of error correction is affected by the number and rules of oral grammar, and the semantic analysis can also change the accuracy of the whole error correction model. The above variables have an obvious linear relationship, and the change curve of correlation coefficient can be obtained according to the change of linear relationship. If the correlation coefficient is close to the value 1 in the variable calculation, the stronger the representativeness is. On the contrary, if the correlation coefficient is close to 0 in the calculation, the direct connection property of the representative variables is weaker. We compare the correlation coefficients between the number of oral grammar and semantic analysis, and the results are shown in Figure 8.

**Figure 9:**
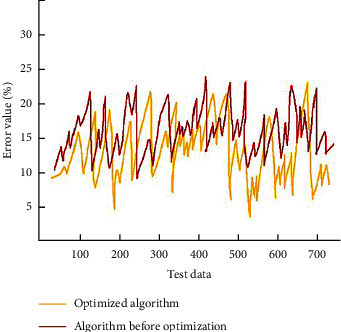
Comparison chart of neural network error before and after optimization.

The results show that the accuracy of semantic analysis for multiple variables can significantly affect the accuracy of the College English oral grammar error correction model. We use the GA-MLP-NN algorithm to support the model and then use modeling tools to divide the network structure. It mainly includes data information collection, training learning model, and prediction results. In order to improve the learning and training speed of the whole model, we design the activation function in the network structure to add the fading layer. According to the training mode of batch processing, the actual data are recorded to minimize the data error of the expected results. Before using GA-MLP-NN, it is necessary to adjust the weight value of participation conditions. The variation of the neural network error peak before and after optimization is shown in Figure 9.

Grammar classroom environment refers to students' perception of classroom physical environment, social environment, and psychological atmosphere, which is between teachers' behavior and learning effect, and becomes an important potential factor that determines learning effect and affects 'students' cognitive and emotional development. Individual explanatory power data from three dimensions of an effective classroom environment show that the learning behavior and situational support have significant explanatory power on the learning effect. However, the data show that the interpersonal support coefficient is not significant. The results are shown in Table 1.

**Figure 10:**
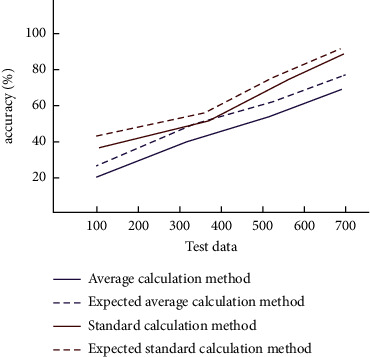
Comparison of results between standard calculation method and average calculation method.

In the analysis of the prediction results of the College English oral grammar error correction model, standard and average methods are used to calculate the error data. The prediction accuracy of the above models was evaluated. If the difference between the output data and the actual data is small, it shows that the accuracy of the prediction results is relatively high. The prediction results of the standard calculation method and average calculation method in the error correction model and the comparison of their expected results are shown in Figure 10.

### 4.1. Analysis of the Results of the Intelligent Image Error Correction Model of College English Spoken Grammar Based on Deep Learning

In oral grammar error correction, relevant algorithms are usually used to compare and estimate the accurate answers, considering that the semantic errors in the original text will have some accuracy impact. We predict the overall degree of correction rate. According to the prediction results, as long as the oral sentence has been corrected, it will be used as a marker. The marked sentence is correct by default, and then the original sentence in the text is deleted. We use the database content as the actual object parameters, mainly from the spoken audio text information in the Internet. There are many kinds of semantic errors in it. Firstly, it is marked manually, and then it is trained by machine learning. We can get the types and locations of grammatical errors by excluding the number information, region information, time information, and other variables. The deep learning algorithm is used to compare the accuracy and recall of the whole model. The comparison results between the original model and the seq2seq model are shown in Table 2.

Thus, the deep learning algorithm can accurately analyze the error location and node in oral grammar and help to improve the accuracy of the error correction model.

## 5. Conclusion

Oral English is the most important communication skill in the process of communication. With the continuous use of College oral English grammar, people pay more and more attention to grammatical errors. At present, the development of deep learning and neural networks is very rapid. The combination of machine learning and artificial neural network improves the overall prediction accuracy and learning speed of the model. Firstly, according to the development status of deep learning and neural networks at home and abroad, this paper analyzes the important role of the above algorithms in machine learning ability and optimization methods. The optimized GA-MLP-NN algorithm is proposed to improve the overall performance in the process of calculating the College English oral grammar error correction model. Secondly, we analyze the internal structure of the basic MLP algorithm. Compared with the traditional multilayer perceptron prediction, the results show that the optimized algorithm significantly improves the operation efficiency of the model and shortens the prediction time. Then, the deep learning model of the whole model is constructed, and the seq2seq model of the deep learning algorithm is used to model the oral grammar error correction system. In the basic model of the comparative modeling process, the accuracy and regression speed of the seq2seq model are significantly improved compared with the traditional model. Finally, the advantages of this model and the variable factors that need to be paid attention to in grammar correction are analyzed. The results show that GA-MLP-NN can change the problem of a huge amount of data and simplify the complexity of the whole model. The deep learning algorithm can improve the error correction accuracy and the overall operation speed of the model.

## Figures and Tables

**Table 1:** The estimated results of the three-dimensional degree of effective classroom environment construction on the learning effect.

Constant	Standard error	Standard coefficient
Learning behavior	1.185	0.512
Interpersonal support	0.082	0.029
Scenario support	0.017	0.102
Learning effect	0.026	0.184

**Table 2:** Comparison between original model and seq2seq model.

Language model	Accuracy (%)	Recall (%)
Original model	25.78	8.54
seq2seq model	30.76	9.68

## Data Availability

The data used to support the findings of this study are available from the corresponding author upon request.
